# The Calponin Family Member CHDP-1 Interacts with Rac/CED-10 to Promote Cell Protrusions

**DOI:** 10.1371/journal.pgen.1006163

**Published:** 2016-07-14

**Authors:** Liying Guan, Xuehua Ma, Jingyan Zhang, Jia-Jia Liu, Yingchun Wang, Mei Ding

**Affiliations:** 1 State Key Laboratory of Molecular Developmental Biology, Institute of Genetics and Developmental Biology, Chinese Academy of Sciences, Beijing, China; 2 University of Chinese Academy of Sciences, Beijing, China; 3 CAS Center for Excellence in Brain Science and Intelligence Technology, Shanghai, China; University of California San Diego, UNITED STATES

## Abstract

Eukaryotic cells extend a variety of surface protrusions to direct cell motility. Formation of protrusions is mediated by coordinated actions between the plasma membrane and the underlying actin cytoskeleton. Here, we found that the single calponin homology (CH) domain-containing protein CHDP-1 induces the formation of cell protrusions in *C*. *elegans*. CHDP-1 is anchored to the cortex through its amphipathic helix. CHDP-1 associates through its CH domain with the small GTPase Rac1/CED-10, which is a key regulator of the actin cytoskeleton. CHDP-1 preferentially binds to the GTP-bound active form of the CED-10 protein and preserves the membrane localization of GTP-CED-10. Hence, by coupling membrane expansion to Rac1-mediated actin dynamics, CHDP-1 promotes the formation of cellular protrusions *in vivo*.

## Author Summary

In response to intra- and extracellular cues, remodeling of the sub-membranous cortical actin cytoskeleton constantly reorganizes the plasma membrane. Thus, distinct types of actin-rich invaginations or protrusions, such as filopodia and lamellipodia, enable cells to explore territory and pull themselves around. Extensive research has shown that the plasma membrane is tightly coupled to the motility machinery. However, how the continuous reorganization of the actin cytoskeleton is coupled with appropriate restructuring of the plasma membrane at the molecular level *in vivo* is unclear. Here, we identified that the single calponin homology (CH) domain-containing protein CHDP-1 promotes the formation of cell protrusions in *C*. *elegans*. CHDP-1 localizes to the cell cortex and through its calponin homology (CH) domain, CHDP-1 directly binds to the master actin regulator Rac1/CED-10 and enhances the membrane localization of the active CED-10 protein. Thus, we discovered a novel CHDP-1/Rac1 module which effectively couples membrane expansion to cortex actin dynamics *in vivo*.

## Introduction

In eukaryotic cells, the actin cytoskeleton forms a cortical shell around the cell periphery, which allows the plasma membrane to undergo frequent structural changes. The Rho family of small GTPases generally act on membranes and affect the movement of these membranes by changing the membrane-associated actin cytoskeleton. Rac1 and Cdc42 induce plasma membrane protrusions, including lamellipodia and filopodia, while RhoA activation specifically stimulates the formation of actin stress fibers [1]. Through the multi-protein WAVE complex, Rac1 indirectly stimulates the formation of new actin filaments [2,3]. Alternatively, Rac1 and CDC42 target PAK family members, which in turn phosphorylate LIMKs (LIM domain kinases), and ultimately act on the actin-severing factor cofilin [4–6]. RhoA often acts as an antagonist of Rac1 and Cdc42, and inhibition of either RhoA or its effector ROCK can result in Rac1 activation [7–9]. The activity of Rho GTPases is strictly controlled by various regulatory proteins, including guanine nucleotide exchange factors (GEFs), which convert the inactive GDP-bound enzymes into active GTP-bound forms, and GTPase-activating proteins (GAPs), which stimulate GTP hydrolysis, thereby converting the active GTPases into their inactive forms.

Cell motility, which involves a continuous reorganization of the cytoskeleton, must be accompanied by appropriate restructuring of the plasma membrane. Indeed, extensive research has identified a diverse array of actin regulatory proteins that are directly associated with the lipid bilayer of the plasma membrane, including vinculin [10,11], talin [12,13], α-actinin [10,14] and cofilin [15]. The plasma membrane is rich in anionic phospholipids, which form electrostatic interactions with cytosolic proteins. Among the structural domains that bind the membrane without specificity for particular phospholipids, the amphipathic helix and Bin/amphiphysin/Rvs (BAR) domains have membrane deformation activity [16,17]. The amphipathic helix domain deforms the membrane by inserting an amphipathic helix or a hydrophobic loop into the inner layer of the plasma membrane and forming additional electrostatic interactions with anionic phospholipids in the cytosolic layer [18]. Proteins in the BAR domain superfamily use their positively charged, curved surface to induce membrane deformation with different degrees of curvature [16]. In particular, the I-BARs (Inverse BAR domains) can deform membranes *in vitro* into protrusion-like shapes [19,20]. However, it is still uncertain whether such membrane deformations actually take place *in vivo*.

Calponin homology (CH) domains have been identified in proteins with a variety of functions ranging from actin binding to signaling [21,22]. The type I and type II CH domains together form the actin-binding region of a large number of F-actin-interacting proteins [23,24]. In contrast, calponin contains a single type III CH domain, which differs markedly from the type I and type II modules. The CH domain in calponin has been implicated in the association of multiple cytoskeleton-related components, but the actin-binding ability of type III CH domains in isolation is questionable [25]. Here, we discovered that the protein CHDP-1, which contains a single type III CH domain and shares homology to calponin in mammals, plays a crucial role in protrusion formation in *C*. *elegans*. With its additional amphipathic helix motif, CHDP-1 localizes to the cell cortex and induces the formation of membrane protrusions. Furthermore, CHDP-1 binds to the GTP-bound active form of the Rac1 homolog CED-10 and facilitates the membrane association of the CED-10 protein. Our work reveals a novel regulatory component which couples membrane expansion to actin dynamics during protrusion formation *in vivo*.

## Results

### Cell protrusion defects in *xd27* animals

**Fig 1:**
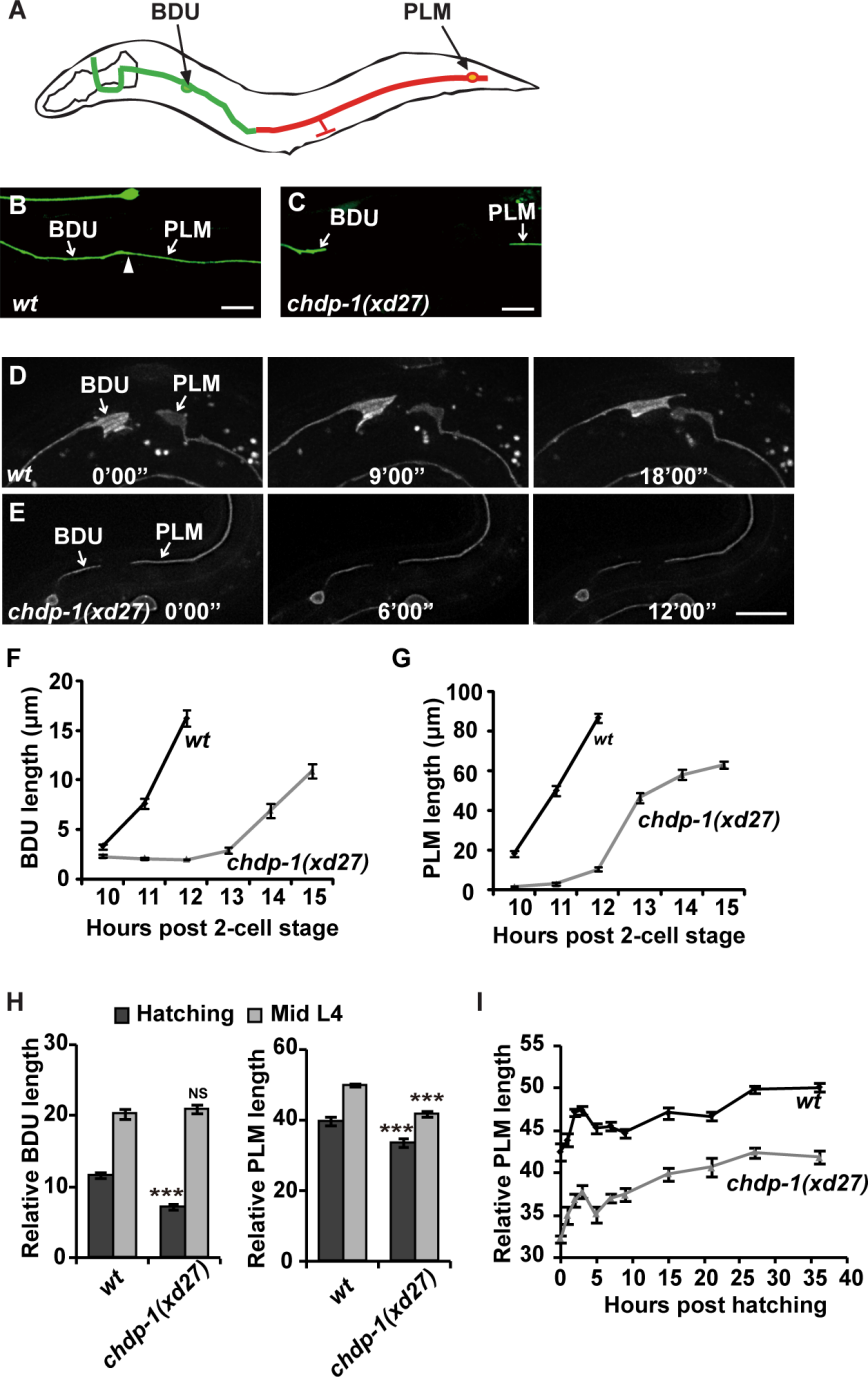
*chdp-1* is required for cell protrusion. (A) Schematic drawing of the BDU-PLM connection. (B) The BDU interneuron connects to the PLM sensory neuron in wild type. The BDU and PLM neurites are indicated by white arrows. The BDU-PLM connecting point is indicated by the white arrowhead. (C) In *chdp-1(xd27)* mutants, the BDU-PLM connection is disrupted. (D) Time-lapse images of P*unc-86*::Myr::GFP in wild type showing the dynamic movements of the BDU and PLM growth cones, which are lacking in *chdp-1(xd27)* animals (E). All scale bars represent 10 μm. (F) BDU and (G) PLM neurite extension during the embryonic stage. (H) Quantification of BDU and PLM length at the embryonic and mid-L4 stages in wild type and *chdp-1(xd27)* mutants. ***p < 0.001; NS, not significant. (I) PLM neurite extension curve in wild-type and *chdp-1(xd27)* animals.

BDU neurons are a pair of interneurons with cell bodies situated laterally in the anterior body of *C*. *elegans*. From its cell body, each BDU neuron projects an anterior process and a posterior process. The PLM neuron has its cell body located in the tail region and sends out an anterior process to the mid-body region of the animal. Our previous work demonstrated that the posterior process of BDU connects with the anterior process of PLM through gap junctions [26] (Fig 1A and 1B). During the formation of the BDU-PLM connection, both the BDU and PLM neurons produce extensively elaborated membrane protrusions to reach each other and finally form the neuronal connection. From a genetic screen to search for mutants which display a BDU-PLM disconnection phenotype, we identified *xd27* (Fig 1C). In *xd27* animals, the cell protrusions on both BDU and PLM cells are greatly reduced (Fig 1D and 1E). Although a single neurite can project out from the posterior BDU or anterior PLM cell bodies in *xd27*, the typical growth-cone-like membrane expansion is missing (Fig 1E). Actin filament dynamics are essential for cell surface protrusion but are not necessary for axonal elongation, which is thought to be mainly mediated by microtubule extension [27]. Intriguingly, the neurites from both BDU and PLM in *xd27* can continuously extend (Fig 1F and 1G). In *xd27* adults, the BDU neurite length is almost indistinguishable from wild type, while the PLM neurite is slightly shorter than wild type (Fig 1H). However, when we followed neurite growth during larval stages, we found that the PLM neurite elongates at a similar speed in both *xd27* and wild-type animals (Fig 1I), suggesting that the formation of actin-mediated cell protrusions may be specifically affected by the *xd27* mutation. In addition to BDU and PLM cells, the head neurons RMED and RMEV, and the D type motor neurons DDs and/or VDs, also display weak neurite extension defects (S1 Fig). However, the locomotion of *xd27* is generally normal. Besides the neural deficits, *xd27* animals display partial embryonic lethality and weak egg laying and distal tip cell migration defects (S1 Fig).

### *chdp-1* is required cell-autonomously for cell protrusion formation

**Fig 2:**
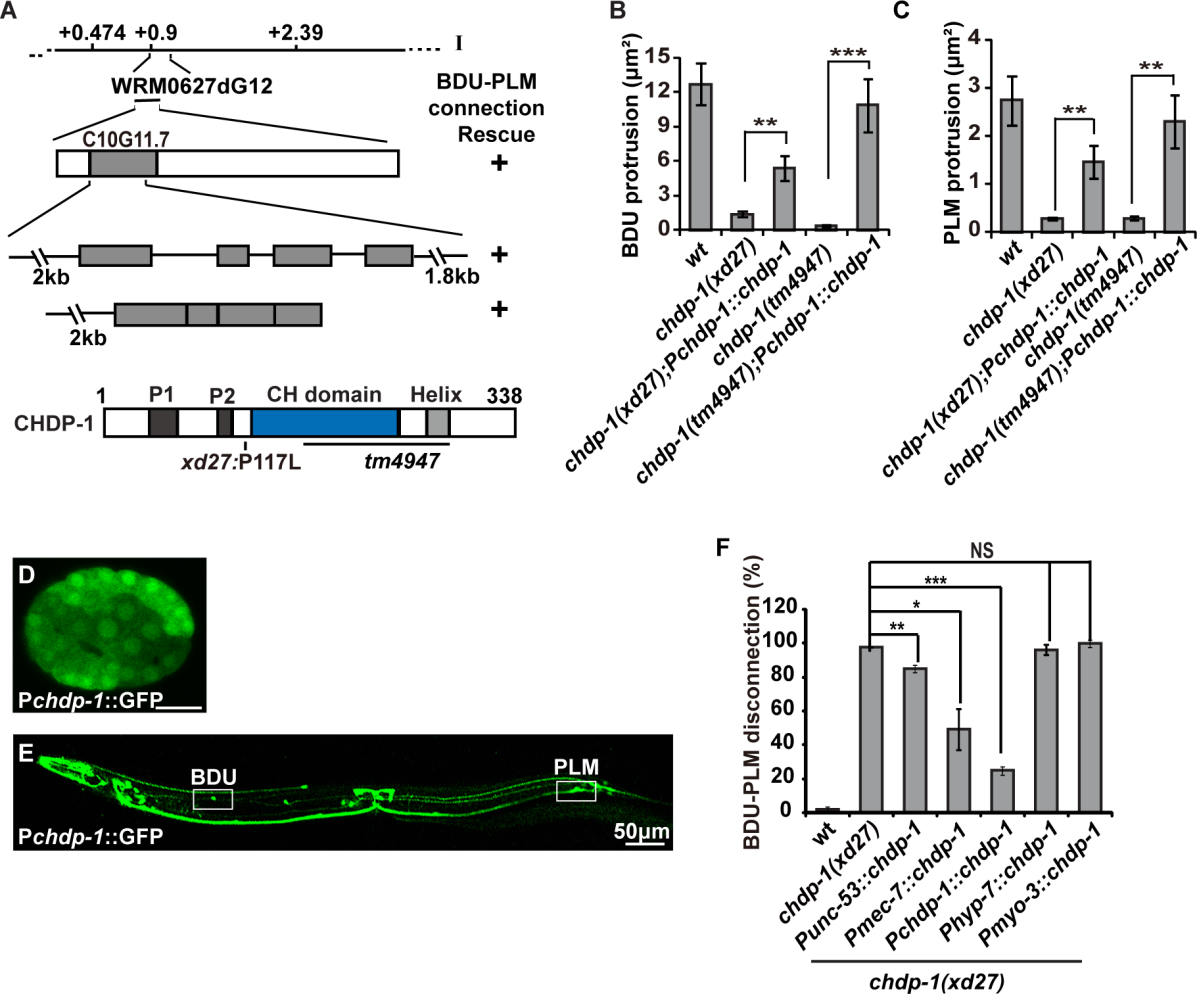
*chdp-1* functions cell-autonomously. (A) The C10G11.7 gene (*chdp-1*) encodes a single CH domain-containing protein. The molecular lesions in *xd27* and *tm4947* are indicated. P1 and P2: proline-rich regions. Helix: amphipathic helix. (B-C) Quantification of BDU (B) and PLM (C) cell protrusion size using P*unc-86*::Myr::GFP in wild type, *chdp-1(xd27)*, *chdp-1(tm4947)*, and the corresponding *chdp-1* rescuing strains. n ≥ 30. (D-E) *chdp-1* gene expression in an embryo (D) and an adult animal (E) revealed by P*chdp-1*::GFP. In the adult, the BDU and PLM cell bodies are boxed. (F) Rescue of the BDU-PLM disconnection phenotype by tissue-specific expression of *chdp-1* using different promoters: P*unc-53* in BDU; P*mec-7* in PLM; P*chdp-1* in both BDU and PLM cells; P*hyp-7* in epidermal cells; P*myo-3* in muscle cells. n ≥ 100. For all quantification analyses, error bars represent the standard error of the mean (SEM); ***p < 0.001; **p < 0.01; *p < 0.05; NS, not significant.

Through genetic mapping and genomic DNA sequencing, we identified C10G11.7 (Fig 2A). C10G11.7 encodes a single type III CH domain-containing protein, which we named CHDP-1 (Fig 2A). It shares sequence similarity with calponin in mammals (S1F Fig). In addition to the CH domain, CHDP-1 contains two proline-rich motifs (P1 and P2) in the N-terminal region and one amphipathic helix motif (Helix) close to the C-terminus (Fig 2A). Proline-rich motifs widely participate in protein-protein interactions [28], while amphipathic helix motifs directly interact with membrane phospholipids [29]. A phenylalanine to leucine change at position 117 was identified in *xd27* worms (Fig 2A). Another *chdp-1* allele, *tm4947*, which removes the amphipathic helix domain and the majority of the CH domain (Fig 2A) and is likely a molecular null, displays a similar membrane protrusion defect to *xd27* (Fig 2B and 2C), suggesting that *xd27* may also act as a strong loss-of-function or null mutation. Introducing a wild-type copy of the *chdp-1* gene into *xd27* or *tm4947* animals restores the proper morphology of developing BDU and PLM cells (Fig 2B and 2C). Thus, mutations of the *chdp-1* gene are indeed responsible for the BDU-PLM connection defect.

The *chdp-1* gene is widely expressed in almost every cell during the early embryonic stage (Fig 2D). From late embryonic to adult stages, however, *chdp-1* expression was gradually restricted to certain sets of neurons including BDU, ALM, AVM, PLM, PVM and PVQ (Fig 2E). When *chdp-1* cDNA was expressed in BDU or PLM alone, or BDU and PLM together, the BDU-PLM connection defect in *chdp-1* mutants was efficiently rescued (Fig 2F). In contrast, expression of *chdp-1* in hypodermis or muscle failed to rescue the BDU-PLM connection defect (Fig 2F), suggesting that *chdp-1* functions cell-autonomously to regulate protrusion formation.

### CHDP-1 localizes to the cell cortex

**Fig 3:**
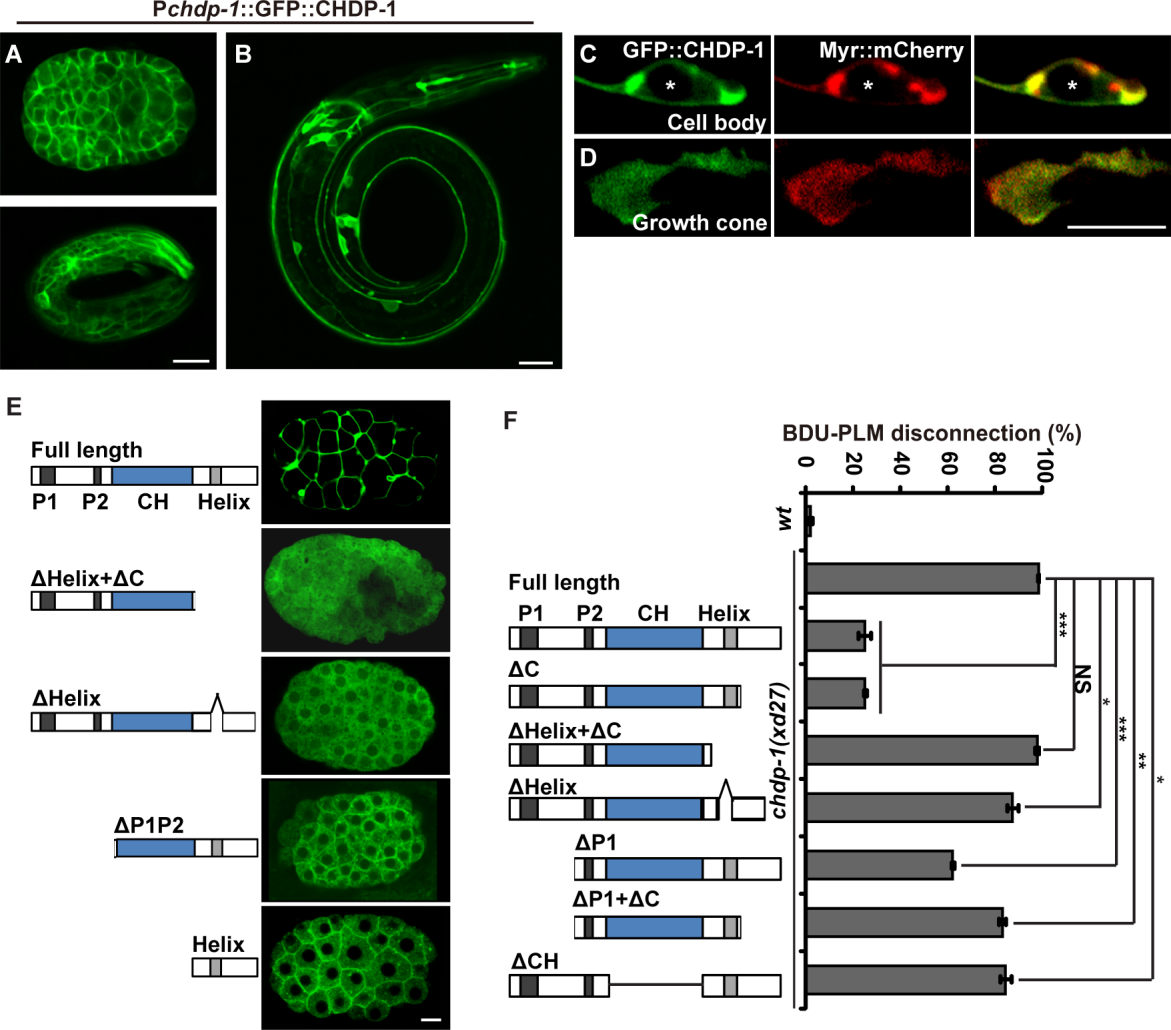
CHDP-1 localizes to and functions at the cell margin. (A) The functional P*chdp-1*::GFP::CHDP-1 reporter localizes to the cell margin during embryonic and larval stage (B). (C-D) GFP::CHDP-1 (expressed using the P*mec-7* promoter) co-localizes with the plasma membrane marker Myr::mCherry (P*mec-7*) in the cell body (C) and the protrusion region (D) of the PLM cell. Asterisks label the nuclei within the cell bodies. (E) Subcellular localizations of truncated CHDP-1 proteins during the embryonic stage. (F) Rescuing activity of different truncated *chdp-1* constructs. n ≥ 100. ***p < 0.001; **p < 0.01; *p < 0.05; NS, not significant.

To understand how CHDP-1 regulates protrusion formation, we examined the subcellular localization of CHDP-1 *in vivo*. A functional translational CHDP-1 fusion green fluorescent protein (GFP) construct was introduced into worms. During the embryonic stage or after hatching, CHDP-1 is distributed specifically at the cell periphery (Fig 3A). After hatching, although some of the GFP::CHDP-1 becomes diffuse in the cytoplasm, most of the signal retains this membrane-like distribution (Fig 3B). Within the PLM neuron, CHDP-1 highlights the cell periphery (Fig 3C), including surface protrusions (Fig 3D). Further co-labeling experiments indicated that CHDP-1 co-localizes well with the plasma membrane marker Myr-mCherry (Fig 3C and 3D), suggesting that the CHDP-1 protein associates with the cell cortex.

The amphipathic helix motif has been implicated in membrane bilayer association [29]. We wondered whether the amphipathic helix motif hooks the CHDP-1 protein onto the plasma membrane. To test this hypothesis, we removed the amphipathic helix motif from CHDP-1 and examined the subcellular localization of the residual CHDP-1 fragment. We found that in the absence of the amphipathic helix motif, the cortex localization of CHDP-1 is completely lost (Fig 3E). In contrast, whenever the amphipathic helix motif is present, the peripheral distribution of CHDP-1 protein is largely preserved (Fig 3E). Thus, the cortex localization property of CHDP-1 is probably mediated by the amphipathic helix motif.

Next, we asked whether the cortex localization is important for CHDP-1 function. To address this question, we removed the amphipathic helix domain and performed rescue experiments. We found that deletion of the amphipathic helix domain largely abolished the rescue effect of CHDP-1 (Fig 3F). Therefore, localization to the cell cortex is crucial for CHDP-1 function.

### CHDP-1 promotes cell protrusion

**Fig 4:**
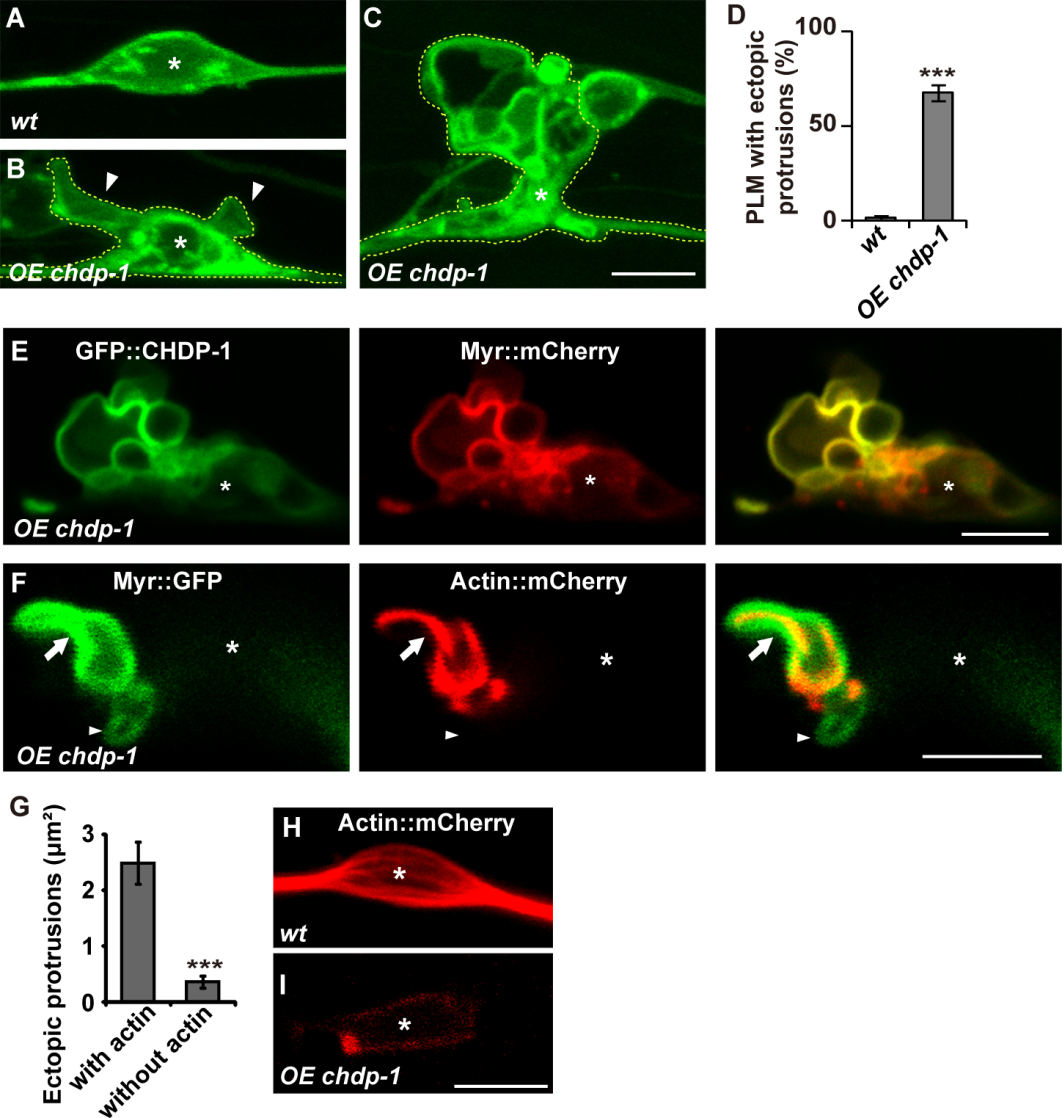
*chdp-1* promotes cell protrusion. (A) The PLM cell body displays a spindle-like cell shape in wild type. (B-C) Ectopic cell protrusions (arrowheads) appear on PLM cells (outlined by yellow dashed lines) when *chdp-1* is over-expressed (*OE chdp-1*). PLM is labeled by P*unc-86*::Myr::GFP. (D) Quantification of PLM cells in wild type and *chdp-1* overexpression animals with ectopic protrusions. (E) GFP::CHDP-1 co-localizes with the plasma membrane marker Myr::mCherry on ectopic protrusions. (F) Some protrusions contain actin (arrows) and some do not (arrowheads). (G) Quantification of protrusion size with or without actin. n ≥ 30. (H-I) The stress-fiber-like structures (H) disappear in *chdp-1* overexpression animals (*OE chdp-1*) (I). On the confocal images, asterisks label the nuclei within cell bodies. Data are presented as mean ± SD; ***p < 0.001.

Without CHDP-1, robust cell protrusion is severely reduced. Furthermore, the amphipathic helix domain has been implicated in membrane deformation [16]. We therefore asked whether CHDP-1 can promote membrane protrusion. To test this possibility, we increased *chdp-1* gene expression in PLM neurons using the P*mec-7* promoter. In wild-type animals, PLM cell bodies generally display smooth spindle-like shapes (Fig 4A). When *chdp-1* gene expression was boosted, additional membrane protrusions of various sizes appeared on the surface of PLM cell bodies (Fig 4B–4D). The P*mec-7* promoter also drives *chdp-1* expression in ALM, AVM, and PVM neurons, and we found that these cells also display ectopic membrane protrusions around the cell body region (S2 Fig). CHDP-1 localizes to the cell cortex. Double staining of CHDP-1::GFP and Myr::mCherry indicated that when it is overexpressed, the CHDP-1 protein still tightly associates with the cortex region (Fig 4E). This suggests that CHDP-1 may function at the cell margin to induce cell protrusion.

The formation of cell surface protrusions is a cooperative result of both membrane expansion and actin cytoskeleton rearrangement. The expanded plasma membrane must be stabilized by the underlying cortical actin network. To address whether the ectopic membrane protrusions are genuine cell surface protrusions, we co-labeled PLM cells with Myr-GFP and mCherry-actin. We found that most of the ectopically expanded plasma membrane is indeed supported by cortical actin (77%, n = 236) (Fig 4F and 4G). However, some protrusions do not contain any detectable underlying actin (23%, n = 236), and these tend to be smaller than those with actin (Fig 4F and 4G). We suspected that these actin-free protrusions may represent newly formed protrusions. Furthermore, in wild-type PLM cell bodies, the actin cytoskeleton is usually organized into stress fiber-like structures (Fig 4H) [30]. However, when ectopic protrusions were induced by *chdp-1* over-expression, most of the actin signal was shifted to the cortex region underlying those ectopic protrusions and no stress fiber-like structures could be found in the center of the PLM cell bodies, where the nucleus is located (Fig 4I). Together, these results suggest that CHDP-1 promotes the formation of cell protrusions *in vivo*.

### CHDP-1 does not bind to actin

**Fig 5:**
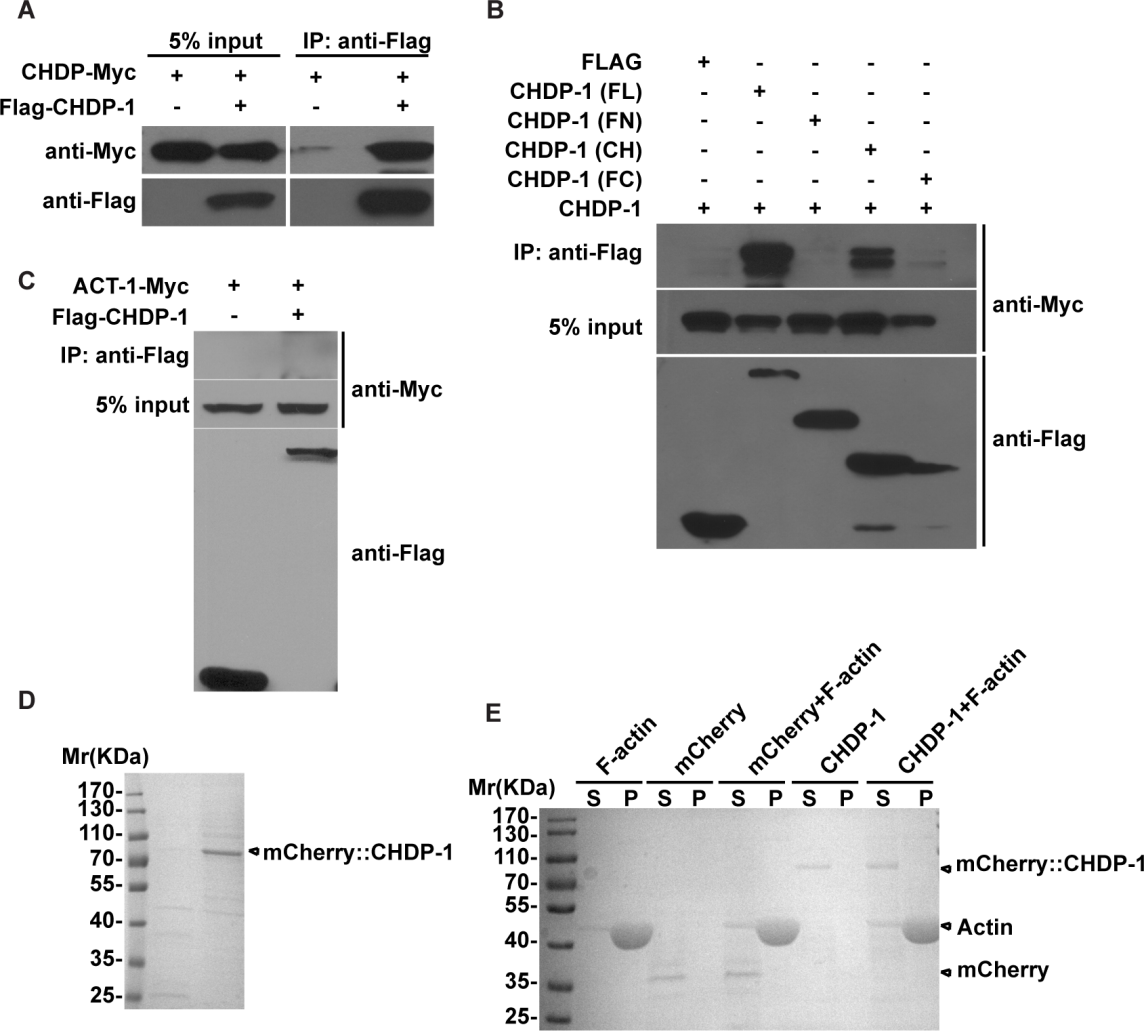
CHDP-1 does not associate with actin. (A) FLAG-tagged CHDP-1 binds to Myc-tagged CHDP-1 in an immunoprecipitation assay. (B) Protein-protein interactions between truncated CHDP-1 fragments in immunoprecipitation assays. FL: full length CHDP-1, FN: N-terminal region, CH: CH domain, FC: C-terminal region. (C) Myc-tagged ACT-1 does not bind to Flag-tagged CHDP-1. (D) Coomassie blue staining of mCherry::CHDP-1, which was purified and resolved on an SDS-PAGE gel. (E) CHDP-1 does not co-sediment with F-actin.

Membrane expansion must be coupled with the actin network to efficiently promote protrusion. Can the cortex-localized CHDP-1 protein associate with actin, thus coupling membrane expansion to actin rearrangement? Interestingly, a tandem array of type I or II CH domains has been shown to possess actin-binding activity [23,24]. Therefore, we tested whether the CHDP-1 protein can self-associate by performing co-precipitation assays on 293T cells transfected with Flag- and Myc-tagged CHDP-1. We found that CHDP-1 molecules can indeed bind to each other (Fig 5A). Truncation analysis indicated that the self-association of CHDP-1 is mediated by the CH domain (Fig 5B). We next tested whether CHDP-1 can bind to actin. We firstly examined whether CHDP-1 binds to monomeric (G) actin. Flag-tagged CHDP-1 did not co-precipitate with worm actin ACT-1 (Fig 5C), suggesting that CHDP-1 does not associate with G-actin. Next, we purified the mCherry::CHDP-1 protein (Fig 5D) and performed co-sedimentation experiments with polymerized filamentous (F) actin. As shown in S2D Fig, the actin motor myosin co-sedimented with F-actin, and this myosin-actin association could be disrupted by ATP. In contrast, CHDP-1 was not sedimented with F-actin at all (Fig 5E). Together, these results indicate that CHDP-1 probably does not promote the formation of cell protrusions through direct actin binding.

### CHDP-1 directly associates with Rac1/CED-10

**Fig 6:**
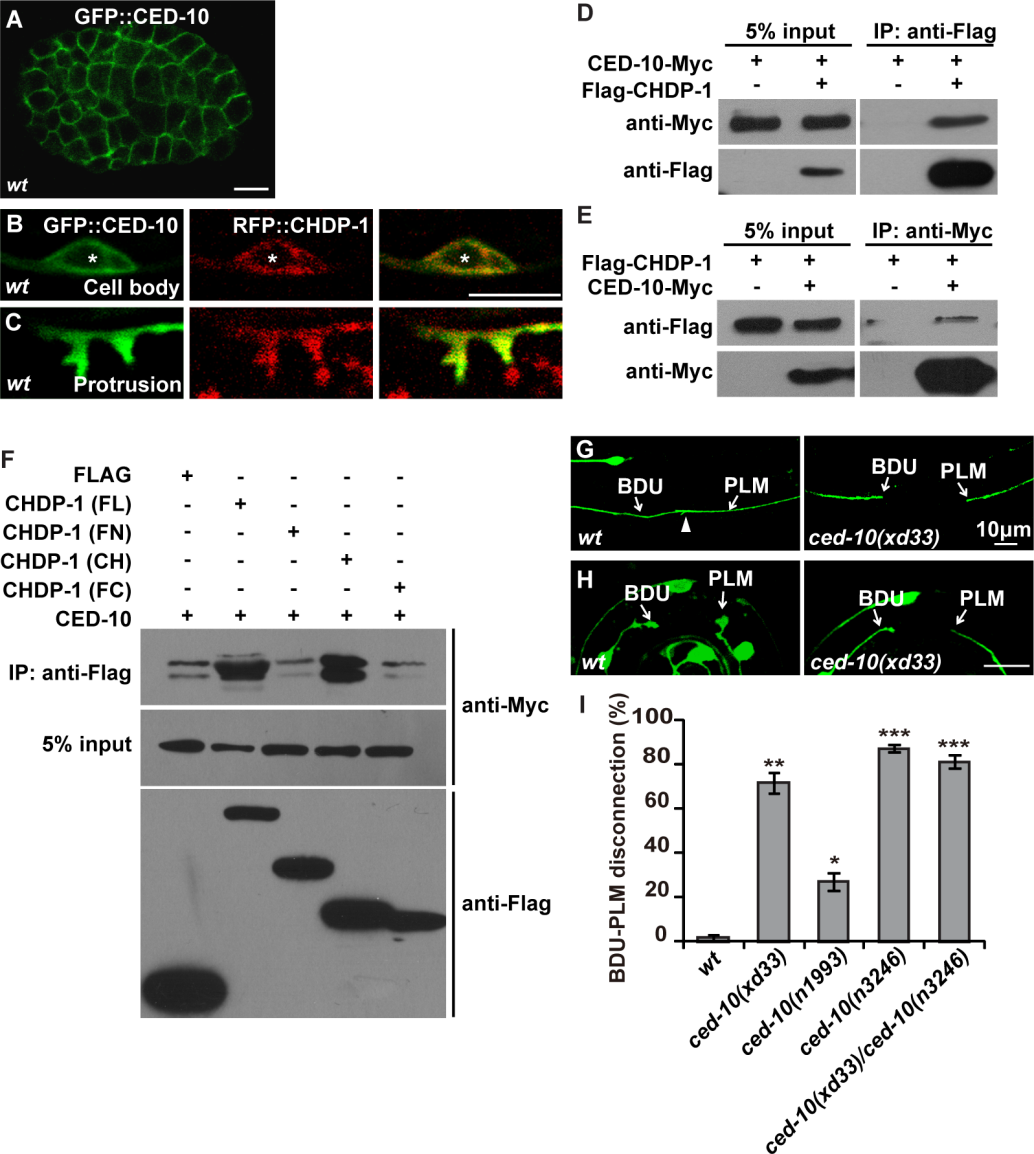
CHDP-1 associates with CED-10. (A) CED-10 localizes to the cell cortex. (B-C) GFP::CED-10 co-localizes with RFP::CHDP-1 at the periphery (C) and in protrusions (D) of the PLM cell. Asterisks label the nucleus within the cell body. (D-E) Myc-tagged CED-10 and Flag-tagged CHDP-1 co-precipitate each other. (F) Protein-protein interactions between truncated CHDP-1 and CED-10 fragments in immunoprecipitation assays. FL: full length CHDP-1, FN: N-terminal region, CH: CH domain, FC: C-terminal region. (G) The BDU-PLM connection is not formed in *ced-10(xd33)* mutants. (H) Membrane protrusion is greatly reduced in *ced-10(xd33)* mutants. (I) Quantification of the BDU-PLM disconnection defect in *ced-10(xd33)*, *ced-10(n1993)*, *ced-10(n3246)*, and *ced-10(xd33)/ced-10(ced-10(n3246)* animals. n ≥ 100. ***p < 0.001; **p < 0.01; *p < 0.05.

To understand how CHDP-1 interacts with the actin cytoskeleton to regulate protrusion formation, we searched for CHDP-1-interacting molecules by performing immunoprecipitation (IP) followed by mass spectrometry (MS). With this approach, we identified the Rho small GTPase family member Rac1/CED-10 [31]. The worm Rac1 homolog, CED-10, is localized to plasma membranes [32] (Fig 6A). In developing PLM neurons, CED-10 co-localizes with CHDP-1 at the periphery of the PLM cell body as well as on the surface of the protrusion region (Fig 6B and 6C). This implies that CHDP-1 and CED-10 may interact with each other at the cell cortex *in vivo*.

To test whether CHDP-1 directly binds to CED-10, we performed the following experiments. Flag-tagged full-length CHDP-1 and Myc-tagged CED-10 were co-expressed in HEK293T cells. After affinity purification, the purified CHDP-1 and CED-10 were mixed together and co-immunoprecipitations were performed with Flag or Myc antibodies. In contrast to the mock-transfected samples, CED-10 was effectively co-precipitated with CHDP-1 (Fig 6D), and the purified CHDP-1 protein was co-precipitated by CED-10 (Fig 6E). Thus, CHDP-1 can directly bind to CED-10. To further identify the domain of CHDP-1 that interacts with CED-10, we performed co-IP experiments using truncated CHDP-1 fragments. We found that the CH domain of CHDP-1 is crucial for the interaction with CED-10 (Fig 6F). Taken together, these results indicate that CHDP-1 may bind to CED-10 through its CH domain.

### CHDP-1 preferentially binds to GTP-CED-10

Coincidently, the BDU-PLM disconnection mutant screen identified a new *ced-10* allele, *xd33* (Fig 6G and 6H). Imaging analysis further indicated that formation of cell protrusions is considerably repressed in *xd33* mutant animals (Fig 6H). *xd33* is a C-to-T missense mutation resulting in a serine 22-to-phenylalanine change (S22F) in CED-10. To understand the role of CED-10 in BDU-PLM connection, we examined two other *ced-10* mutants, *n1993* and *n3246* and found that they both display similar BDU-PLM disconnection defects to *xd33* (Fig 6I). Previous report showed that the *n3246* mutation is recessive to wild type, but homozygote *ced-10(n3246)* animals display defects not seen in *ced-10*(*n1993)* [33]. In *xd33/n3246* trans-heterozygote animals, we found that BDU-PLM disconnection defect is similar to that of *xd33* homozygote. Furthermore, when the wild-type *ced-10* gene was introduced into *xd33* animals, the BDU-PLM connection could be largely restored (S3 Fig), suggesting that the loss-of-function of *ced-10* is responsible for the membrane protrusion defect in the corresponding *ced-10* mutant worms. We further constructed *chdp-1(xd27);ced-10(xd33)* and *chdp-1(tm4947);ced-10(xd33)* double mutants and found that those double mutant animals displayed similar degree of BDU-PLM disconnection defect as *chdp-1(xd27)* animals (S3 Fig), implying that *chdp-1* may function together with *ced-10* to regulate cell protrusion.

**Fig 7:**
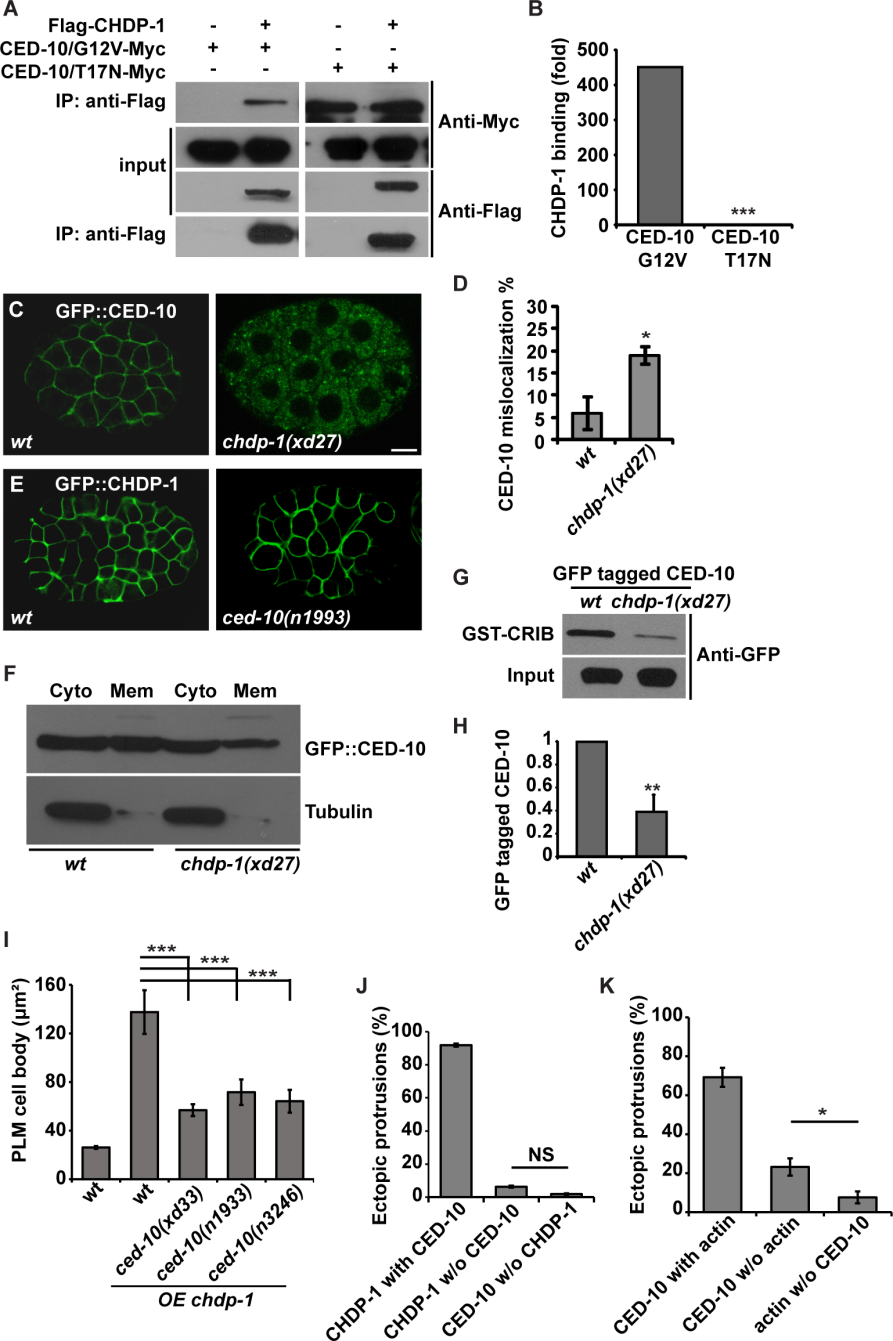
CHDP-1 promotes the membrane localization of CED-10. (A) Immunoprecipitation assay of FLAG-tagged CHDP-1 with Myc-tagged CED-10 (G12V), which mimics active GTP-bound CED-10, and Myc-tagged CED-10 (T17N), which mimics inactive GDP-bound CED-10. CHDP-1 binds more strongly to the G12V variant than the T17N variant. (B) Quantification of the relative level of binding between FLAG-CHDP-1 and CED-10 (G12V) or FLAG-CHDP-1 and CED-10 (T17N). ***p < 0.001. (C) The membrane localization of CED-10 is defective in *chdp-1(xd27)* mutant animals. (D) Quantification of the CED-10 localization defect in wild type and *chdp-1(xd27)* animals. n ≥ 50. (E) The membrane localization of GFP::CHDP-1 is not altered in *ced-10(n1993)* mutants. (F) In a cell fractionation assay, the amount of GFP::CED-10 on the plasma membrane is decreased in *chdp-1(xd27)* mutants. (G) A GST-fused CRIB domain of PAK binds active GTP-CED-10. The amount of GFP tagged CED-10 pulled down by CRIB is decreased in *chdp-1(xd27)* animals. (H) Quantification of the amount of GFP-CED-10 pulled down by GST-CRIB in wild type and *chdp-1(xd27)*. (I) The extensive ectopic cell protrusion is significantly reduced in *ced-10(xd33)*, *ced-10(n1933)* and *ced-10(n3246)* animals. (J) The co-distribution of RFP::CHDP-1 and GFP::CED-10 on ectopic PLM protrusions. (K) The co-distribution of mCherry::actin and GFP::CED-10 on ectopic PLM protrusions. Data are presented as mean ± SD; ***p < 0.001, **p < 0.01, *p < 0.05.

As a member of the Rac family of GTPases, Rac1 cycles between the inactive GDP-bound form and the active GTP-bound form. We then asked how CHDP-1 interacts with different forms of CED-10. The glycine to valine mutation at position 12 (G12V), which is canonical for constitutive activation of Ras superfamily GTPases, mimics the GTP-bound active state. In contrast, the threonine to asparagine mutation at position 17 (T17N) mimics the GDP-bound inactive state. Co-immunoprecipitation experiments with these two mutant forms of CED-10 showed that the CHDP-1 protein precipitates much higher levels of the G12V mutant than the T17N mutant (Fig 7A and 7B). This suggests that CHDP-1 preferentially associates with GTP-bound active CED-10.

### CHDP-1 facilitates the cell cortex localization of CED-10

Both CHDP-1 and CED-10 localize to the cell margin. How does the direct association of CHDP-1 contribute to regulation of CED-10 function? In *chdp-1* mutant animals, we found that the cortex localization of CED-10 is partially lost (Fig 7C and 7D). In contrast, localization of CHDP-1 to the cell margin remains intact in various *ced-10* mutants, including *ced-10(xd33)*, *ced-10(n1993)* and *ced-10(n3246)* (Fig 7E and S3 Fig). Cell-fractionation assays further indicated that the proportion of membrane-bound CED-10 is reduced in *chdp-1* mutants compared with wild-type samples (Fig 7F). The reduction of membrane-bound CED-10 suggested that the level of GTP-bound, active CED-10 protein maybe decreased in *chdp-1* mutants. Therefore, we further tested the GTP-CED-10 level *in vivo*. Active GTP-Rac1 binds to the CRIB (Cdc and Rac interactive binding) domain of its effector PAK, while inactive GDP-Rac1 does not [34]. Thus, we performed pull-down assays on the PAK CRIB domain. In contrast to wild type, we found that the amount of GFP-tagged CED-10 pulled down by the PAK CRIB domain was considerably decreased in *chdp-1* lysates (Fig 7G and 7H). Together, these data suggested that CHDP-1 facilitates the cell margin localization of CED-10 *in vivo* by stabilizing the active GTP-bound CED-10 protein.

### CED-10 is required for CHDP-1-induced cell protrusion

As the critical regulator of actin dynamics, is CED-10 required for CHDP-1-induced cell protrusion? We showed that over-expression of CHDP-1 leads to extensive cell protrusion in PLM cell bodies. In three different *ced-10* mutants, however, the ectopic protrusion on the surface of PLM cell bodies is significantly reduced. We measured the size of the PLM cell body (Fig 7I). In wild-type animals, the maximum area of a PLM cell body is about 26 μm^2^. In *chdp-1* over-expression animals, the area of the PLM cell body is expanded to around 138 μm^2^. In *ced-10* mutant animals, the area of the PLM cell body is 57 μm^2^ (*xd33*), 72 μm^2^ (*n1933*) and 64 μm^2^ (*n3246*) respectively (Fig 7I).

We then examined how CED-10 coordinates with CHDP-1 on the ectopic protrusions. After co-injecting *mec-7* promoter-driven *rfp*::*chdp-1* and *gfp*::*ced-10* constructs into wild-type animals, we found that most, if not all, CHDP-1 and CED-10 are co-distributed on the ectopic cell protrusions (Fig 7J). Very few protrusions contain either CHDP-10 (6.3%) or CED-10 (1.9%), suggesting a tight association of CHDP-1 and CED-10 on those ectopic protrusions. Next, we tested whether the previously described “actin-free” protrusions contain CED-10 or not. Co-labeling of PLM cells with mCherry::ACT-1 and GFP::CED-10 showed that while the majority of protrusions (69.2%) contain both CED-10 and actin, a significant proportion (23.2%) contain CED-10 without actin (Fig 7K). Thus, CED-10 is likely more closely associated with CHDP-1 than with actin to mediate protrusion formation (S3 Fig).

## Discussion

Coupling membrane expansion to actin filaments dynamics is essential for formation of membrane protrusions. In this report, we identified that the calponin-like protein CHDP-1 promotes membrane protrusion and associates with the Rac1/CED-10 GTPase in *C*. *elegans* (S3 Fig).

Two mechanisms of membrane deformation have been linked to the direct binding of proteins to the membrane. One utilizes the electrostatic interactions between the lipid-binding domains on the protein surface and the negatively charged lipids in the membrane. An outstanding example of this class of lipid-binding domain is the BAR domain. BAR domains usually form dimers and the lipid-binding surface of BAR has intrinsic curvature [35]. In particular, the I-BAR family, with its cigar-shaped dimers, can promote negative curvatures, which are frequently found inside plasma membrane protrusions [36]. In addition to interacting electrostatically with lipids, BAR proteins can also recruit other proteins, which may further modify the membrane deformation. IRSp53 (insulin receptor phosphotyrosine 53 kDa) consists of an N-terminal I-BAR domain, followed by a partial CRIB domain and an SH3 domain. Through the CRIB domain, IRSp53 binds to Cdc42 [37], which regulates the formation of filopodia in mammalian cells [38,39]. Through its SH3 domain, IRSp53 also associates with a set of actin regulatory factors, including WAVE1, WAVE2, Mena, mDia1, dynamin, and N-WASp [40]. Thus, IRSp53 allows tight coupling of membrane protrusions to actin dynamics. Direct protein insertion into the membrane is another mechanism used to alter membrane curvature. This mechanism of membrane deformation was first characterized for proteins containing an amphipathic helix; the cylindrical helix lies parallel to the membrane, exposing its hydrophilic surface to the cytosol [41]. However, it is unlikely that a single molecule or a single dimer could act alone to deform the membrane. The assembly of many molecules at the same place on the membrane may be required for deformation. Indeed, in bacteria, the peripheral membrane protein MinD, which contains a highly similar amphipathic helix to CHDP-1, forms polymers and the polymerization of MinD proteins enhances the membrane affinity of the single amphipathic helix domain [29]. Given the fact that CHDP-1 molecules can associate with each other, it is a reasonable proposal that the assembly of chains of CHDP-1 molecules may create sufficient membrane curvature to deform the plasma membrane.

What is the biological significance of the enlarged plasma membrane surface? During the formation of neuronal connections, the distal tips of axons extend highly dynamic membrane expansions, the growth cones, to actively search for environmental cues that will guide them toward their proper target cells [27]. We previously showed that Wnt signaling is involved in BDU-PLM connection [26]. Therefore, it is possible that CHDP-1 promotes the formation of cell protrusions in order to increase the sensitivity of BDU and PLM neurons to detect Wnt signals. Interestingly, the expression of *chdp-1* in either BDU or PLM alone significantly rescued the BDU-PLM disconnection defect in *chdp-1* mutant animals, suggesting that the expanded growth cone of a given neuron may provide sufficient signal detection capacity to locate its target cell during the connection process. Given the fact that BDU and PLM neurites can elongate towards each other in both wild-type and *chdp-1* mutant animals, we suspect that the growth cone protrusion may be specifically required for the final short-range extension of the neurite tip, but is not necessary for long-distance travel of a neurite. It is currently unclear whether similar principles apply to other neuronal connections *in vivo*.

How does CHDP-1 couple membrane protrusion to the actin cytoskeleton? CHDP-1 contains a single CH domain, which shares similarity to that in the mammalian calponin family. In the phylogenetic tree of CH domain-containing proteins, calponins form a separate branch together with other proteins that possess a single CH domain (Vav, IQGAP, ARH-GEF6, and SM22) [23]. Another calponin homolog, CPN-1 (CaPoNin-1), can partially fulfill the functional requirement for CHDP-1 in BDU-PLM connection (reducing the frequency of disconnection from around 95% in *chdp-1(xd27)* mutant animals to 60%), suggesting that CHDP-1 is likely also derived from an ancestral protein in *C*. *elegans*. The isolated CH domains of calponin family proteins fail to associate with F-actin [25]. Instead, they bind to a variety of cytoskeleton and signaling components, including tubulin, intermediate filament, ROCK (Rho-associated kinase), ERK1, and ERK2 [25]. It has therefore been suggested that calponin functions as a scaffolding protein for cytoskeletal structures and/or adaptor molecules in signaling pathways. Here, we found that the CH domain of CHDP-1 does not bind to either G- or F-actin, but instead binds to the Rac1 homolog CED-10. This suggests that CHDP-1 may influence actin cytoskeleton dynamics by directly regulating Rac1/CED-10. Intriguingly, CHDP-1 preferentially associates with GTP-bound active CED-10. In addition, the plasma membrane localization of CED-10 is impaired in the absence of CHDP-1. Coincidently, expression of constitutively active CED-10 in *C*. *elegans* promotes extensive branching [42], suggesting that Rac1 plays an important role in actin polymerization. Another interesting observation is that CHDP-1 molecules may associate with each other also through the CH domain. We suspect that the assembly of chains of CHDP-1 molecules may create sufficiently large membrane protrusions, as well as providing anchor sites for numerous CED-10 molecules, which will efficiently recruit cortical actin to stabilize the protrusions. Thus, CHDP-1 promotes plasma membrane protrusion and stabilizes the expanded membrane by promoting actin cytoskeleton rearrangement through interaction with Rac1/CED-10.

## Materials and Methods

### *C*. *elegans* genetics

Culture and manipulation of *C*. *elegans* strains were performed using standard methods. Mutants used in this studies are: LGI, *chdp-1(xd27)*, *chdp-1(tm4947)*; LGII, *juIs76(Punc-25*::*GFP)*; LGIV, *ced-10(n1993)*, *ced-10(xd33)*, *ced-10(n3246)*, *kyIs262*(P*unc-86*::MYR::GFP, P*odr-1*::dsRed). The *xd27* and *xd33* mutations were isolated from *kyIs262* (P*unc-86*::Myr::GFP) animals treated with EMS. A total of 2,500 mutagenized haploid genomes were screened. The isolated strains were outcrossed with the N2 strain at least four times.

### Generation of transgenic lines and constructs

Corresponding *chdp-1* DNA fragments were amplified from N2 genomic DNA to perform the *chdp-1* cloning experiment. *chdp-1* cDNA was amplified by reverse transcription. *ced-10* cDNA was obtained from Dr. Yuji Kohara. GFP sequence was inserted into P*chdp-1*::CHDP-1 constructs. For protein co-localization, the RFP fragment was inserted into the P*mec-7*::CHDP-1 construct. The *mec-7* promoter was inserted into P*ced-10*::GFP::CED-10 construct to replace the *ced-10* promoter. In general, plasmid DNAs of interest were injected at 10-50ng/μl and the co-injection markers P*odr-1*::RFP, *rol-6* or P*odr-1*::GFP were injected at 10-50ng/μl. The corresponding transgenes and constructs are listed in (S1 Table).

### Image collection and phenotypic quantification

Animals were mounted on 2% agar pads in M9 buffer containing 1% 1-phenoxy-2-propanol and examined by fluorescence microscopy unless indicated otherwise. Fluorescence photographs were taken using a Zeiss Axioimager A1 with an AxioCam digital camera and Axiovision rel. 4.6 software (Carl Zeiss) or an IX81 Olympus inverted confocal microscope. Contact was considered defective if the BDU neurite failed to touch the PLM neurite. Neurite and worm body lengths were measured with NIH ImageJ software. The length of each neurite was traced from the center of the cell body to the tip of neurite. The length of each animal was measured from the center of the PLM cell body to the tip of the nose. The relative BDU or PLM neurite length is defined as neurite length/animal length. The PLM cell body was measured using ImageJ software. To measure the co-distribution of actin, CED-10 and CHDP-1 on ectopic protrusions, fluorescence photographs were taken using confocal microscopy. The size of individual protrusions was measured with NIH Image J software using the line-tracing tool. All data are shown as mean ± SD. Statistical analyses were performed with Student’s t-test. For each genotype, more than 20 animals were analyzed.

### Quantification of membrane protrusions

At different time points, eggs were mounted on thin 1% agar pads in M9 buffer; gentle pressure was applied to crack the egg shells, and embryos were examined immediately by fluorescence microscopy. To measure the membrane protrusion area, newly hatched L1 animals were collected and photographs were taken within 10 min of hatching. The expansion area was defined by linking the first branching or elaboration site of the neurite with the tips of all the branches or filopodia. The area was measured with NIH ImageJ software.

### Quantification of membrane localization of CED-10

Some *chdp-1(xd27)* embryos die at the later elongation stage. Therefore we dissected gravid adults and obtained embryos at early developmental stages (from the 16-cell stage to gastrulation stage). Then the collected embryos were mounted on thin 1% agar pads in M9 buffer and examined immediately by fluorescence microscopy. Embryos in which at least half of the cells contained diffuse GFP::CED-10 or GFP::CHDP-1 signal were considered as showing defective localization of the GFP::CED-10 or GFP::CHDP-1 marker. All images were collected using Zeiss Axioimager A1 with an AxioCam digital camera and Axiovision rel. 4.6 software (Carl Zeiss). For each sample, at least 50 embryos were scored.

### Time-lapse imaging

Newly hatched L1 animals were anesthetized with 0.1mM levamisole in M9 buffer and mounted on 2% agar pads at 22°C. Time-lapse images were captured every 1.5 min using a Delta-vision Core imaging system (Applied Precision) with an UPLSApo 100 6/1.40NAoil-immersion objective and a Photometrics CoolSnap HQ camera. Deconvolution and analysis of images were performed with Softworx (Applied Precision).

### Immunoprecipitation for mass spectrometry analysis

Worm lysates were prepared from the N2 strain carrying either *xdIs23*(P*chdp-1*::GFP) or *xdIs48*(P*chdp-1*::GFP::CHDP-1). Specifically, worms were collected and lysed with a Dounce homogenizer (Cheng-He Company, Zhuhai, China) [43] in pre-chilled homogenizing buffer (50mM Tris-Cl pH8.0, NaCl 150mM, 0.5% sodium deoxycholate, 1% Triton-X 100, protease inhibitor [Roche]), then incubated for 15 min on ice, and centrifuged at 12,000 rpm for 15 min at 4°C. The supernatants were incubated with GFP polyclonal antibody (Rabbit, Abcam, ab290, 1:1,000 dilution) overnight, and then incubated with protein A agarose beads (Cat#17-0780-01, GE) for 4 hr at 4°C. The pellet was washed three times in washing buffer (50mM Tris-Cl pH8.0, 150mM NaCl, 1% NP-40, 1 mM PMSF), and then boiled in sample buffer for 5 min. The boiled samples were resolved by SDS-PAGE gel and protein bands of interest were excised from SDS-PAGE gel and in-gel digestion was performed using a well-established protocol with slight modifications [44]. Briefly, the proteins embedded in gel slices were reduced with 10mM DTT and alkylated with 55mM iodoacetamide, and then digested overnight with sequencing grade trypsin (Sigma, USA) at 37°C. The tryptic peptides were analyzed by mass spectrometry using a TripleTOF 5600 mass spectrometer (AB SCIEX, Canada) coupled to an EksigentNanoLC. Peptide identification and quantification were performed with the ProteinPilot 4.2 software (AB SCIEX). The *C*. *elegans* proteome sequences (Uniprot) was used as the database and the mass tolerance was set to 0.05 Da for the database search. The false discovery rate (FDR) analysis was performed using the software PSPEP integrated with ProteinPilot. About 10 cytoskeleton-related proteins were identified in the mass spectrometry analysis. GST pull-down and co-immunoprecipitation assays were then performed to verify the putative protein-protein interactions. Among the tested proteins, CED-10 displayed consistent CHDP-1-binding activity in both GST pull-down and co-immunoprecipitation assays.

### Co-immunoprecipitation

cDNA fragments were amplified and cloned into modified pcDNATM3.1/myc-HIS(-) or pFLAG-CMV-2 vectors through standard procedures. HEK293T cells were cultured in DMEM medium supplemented with 12% FBS. Plasmid transfections were carried out using Lipofectamine 2000 (Invitrogen). 24 hours after transfection, cells were harvested and lysed for 30 min at 4°C. After centrifugation, the supernatants were incubated with anti-FLAG M2 affinity gel beads at 4°C overnight and then washed three times with washing buffer and incubated with SDS-sample buffer. Samples were resolved by standard immunoblotting techniques. To quantify the binding affinity between CHDP-1 and different CED-10 mutant proteins (G12V and T17N mutant forms), the absolute signal intensity of individual protein bands was analyzed with ImageJ. The relative protein level was achieved by subtracting the respective FLAG control. For binding experiments with truncated CHDP-1, the following fragments were used: FN (AA 1–146), CH (AA 121–242) and FC (AA 243–338). All the co-immunoprecipitation experiments were repeated at least three times.

### F-actin sedimentation

The Actin Binding Protein Spin-Down Biochem Kit (Cat. # BK001) from Cytoskeleton Inc was used to detect the F-actin binding activity of CHDP-1. The recombinant proteins Flag::mCherry::CHDP-1 and Flag::mCherry were expressed in HEK293FT cells. 24 hours after transfection, cells were harvested and lysed for 30 min at 4°C. After centrifugation, the corresponding supernatants were incubated with anti-FLAG M2 affinity gel beads at 4°C for purification of tagged proteins. F-actin was prepared from muscle actin and suspended in general actin buffer at room temperature for 1 hour. Proteins at a molar ratio of 1:3 (recombinant protein: F-actin) were incubated at 25°C for 30 minutes and pelleted at 15 0000g for 1.5 hours. Subsequently, protein samples were resolved by SDS-PAGE gel. The muscle myosin was used as a positive control for F-actin binding assay.

### Cell fractionation

Wild-type and *chdp-1(xd27)* worms expressing P*ced*-10::GFP::CED-10 were collected and washed in M9 buffer. Then 500μl lysis buffer (250 mM sucrose, 50 mM Tris–HCl pH6.8, 1 mM EDTA) were added and the samples were homogenized with a Dounce homogenizer (Cheng-He Company, Zhuhai, China) (Chen et al., 2010) on ice for 15 min. The nuclear pellet was removed by centrifuging at 3,000 rpm for 10 min at 4°C. The supernatant was further centrifuged at 40,000 rpm for 1 hr. The new supernatant was collected as the cytosolic fraction. The pellet was further washed and centrifuged at 40,000 rpm for 45 min to obtain the membrane fraction. All samples were boiled with 2xSDS loading buffer then subjected to 10% SDS-PAGE gel analysis.

### PAK-RBD pull-down assay

PAK-GST protein beads (human P21 activated kinase PBD) were used to detect the GTP-bound form of CED-10. Worm lysates were prepared from wild type and *chdp-1(xd27)* mutant animals carrying P*ced-10*::GFP::CED-10. Worms were lysed in pre-chilled homogenizing buffer with a Dounce homogenizer (Cheng-He Company, Zhuhai, China) and incubated for 15 min on ice, then centrifuged at 12,000 rpm for 15 min at 4°C. The supernatants were incubated with PAK-GST protein beads (Cat#PAK02, Cytoskeleton Inc) for 4 hr at 4°C. The pellet was washed three times and then boiled in sample buffer for 5 min. The boiled samples were resolved by SDS-PAGE and detected with anti-GFP antibody. The pull-down experiments were repeated three times. The absolute intensity of each protein band was quantified with ImageJ. The relative protein level was determined by normalizing each sample with the corresponding input, and then normalizing each *chdp-1(xd27)* sample with the paired WT sample.

## Supporting Information

S1 Fig Other neural defects in *chdp-1(xd27)* animals. (A) RMED and REMV neurites in *xd27* animals are shorter than those in wild-type animals. (B) Quantification of the shorter RMED/V neurite phenotype in wild-type and *xd27* animals. (C) Gaps appear on DD and/or VD neurons in *xd27* animals. (D) Quantification of DD and VD gaps in wild-type and *xd27* animals. (E) Protein sequence alignment of CH domains in representative calponin family members. Ce, *Caenorhabditis elegans*; Dm, *Drosophila melanogaster*; Hs, *Homo sapiens*; Sp, *Schizosaccharomyces pombe*.
(TIF)

S2 Fig CHDP-1 promotes cell protrusion. (A) Ectopic cell protrusions appear on ALM cells when *chdp-1* is over-expressed (*OE chdp-1*). (B) Ectopic cell protrusions appear on PVM cells when *chdp-1* is over-expressed (*OE chdp-1*). (C) Ectopic cell protrusions appear on AVM cells when *chdp-1* is over-expressed (*OE chdp-1*). ALM, PVM and AVM are labeled by P*unc-86*::Myr::GFP. (D) The actin motor myosin co-sediments with F-actin and this myosin-actin association is disrupted by ATP.
(TIF)

S3 Fig CHDP-1 interacts with CED-10 to mediate protrusion formation. (A) Quantification of BDU-PLM connection defects in wild type and *ced-10(xd33)* mutants, and the corresponding rescuing strains. n ≥ 100. (B) Quantification of BDU-PLM connection defects in wild type, *chdp-1(xd27)*, *chdp-1(tm4947)*, *ced-10(xd33)* and the corresponding double mutant strains. n ≥ 100. (C) The membrane localization of GFP::CHDP-1 is not altered in *ced-10(xd33)* and *ced-10(n3246)* mutants. (D) Schematic drawing of the role of CHDP-1 and CED-10 in protrusion formation.
(TIF)

S1 Table Transgenes and strains generated in this study. (DOCX)
