# Supplementary figures and images for: The Calponin Family Member CHDP-1 Interacts with Rac/CED-10 to Promote Cell Protrusions
Source: PLoS Genet. 2016 Jul 14;12(7):e1006163. doi: 10.1371/journal.pgen.1006163 (PMC4944944; doi:10.1371/journal.pgen.1006163)

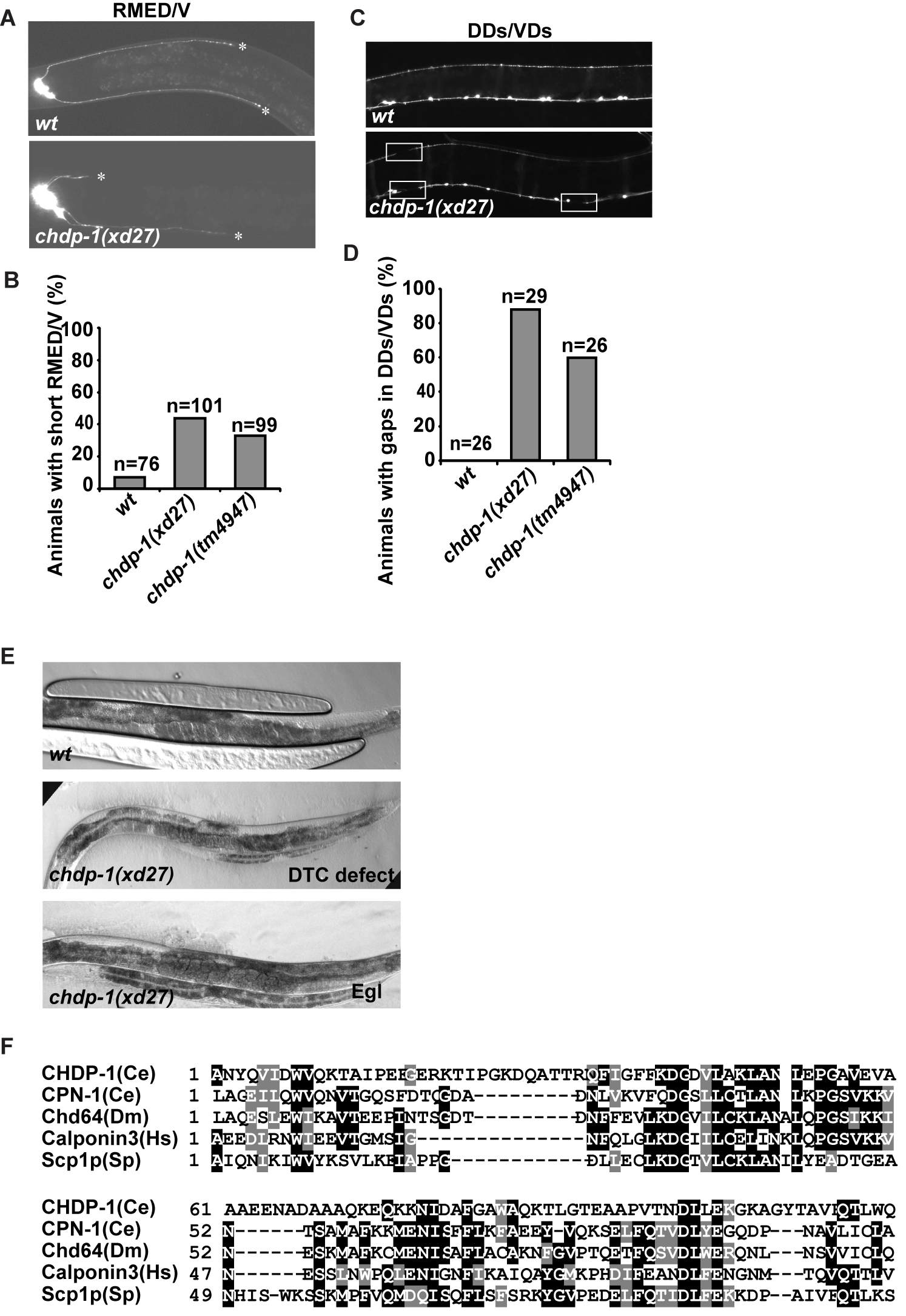

Supplement: S1 Fig — (A) RMED and REMV neurites in xd27 animals are shorter than those in wild-type animals. (B) Quantification of the shorter RMED/V neurite phenotype in wild-type and xd27 animals. (C) Gaps appear on DD and/or VD neurons in xd27 animals. (D) Quantification of DD and VD gaps in wild-type and xd27 animals. (E) Protein sequence alignment of CH domains in representative calponin family members. Ce, Caenorhabditis elegans; Dm, Drosophila melanogaster; Hs, Homo sapiens; Sp, Schizosaccharomyces pombe. (TIF) [file pgen.1006163.s001.tif]

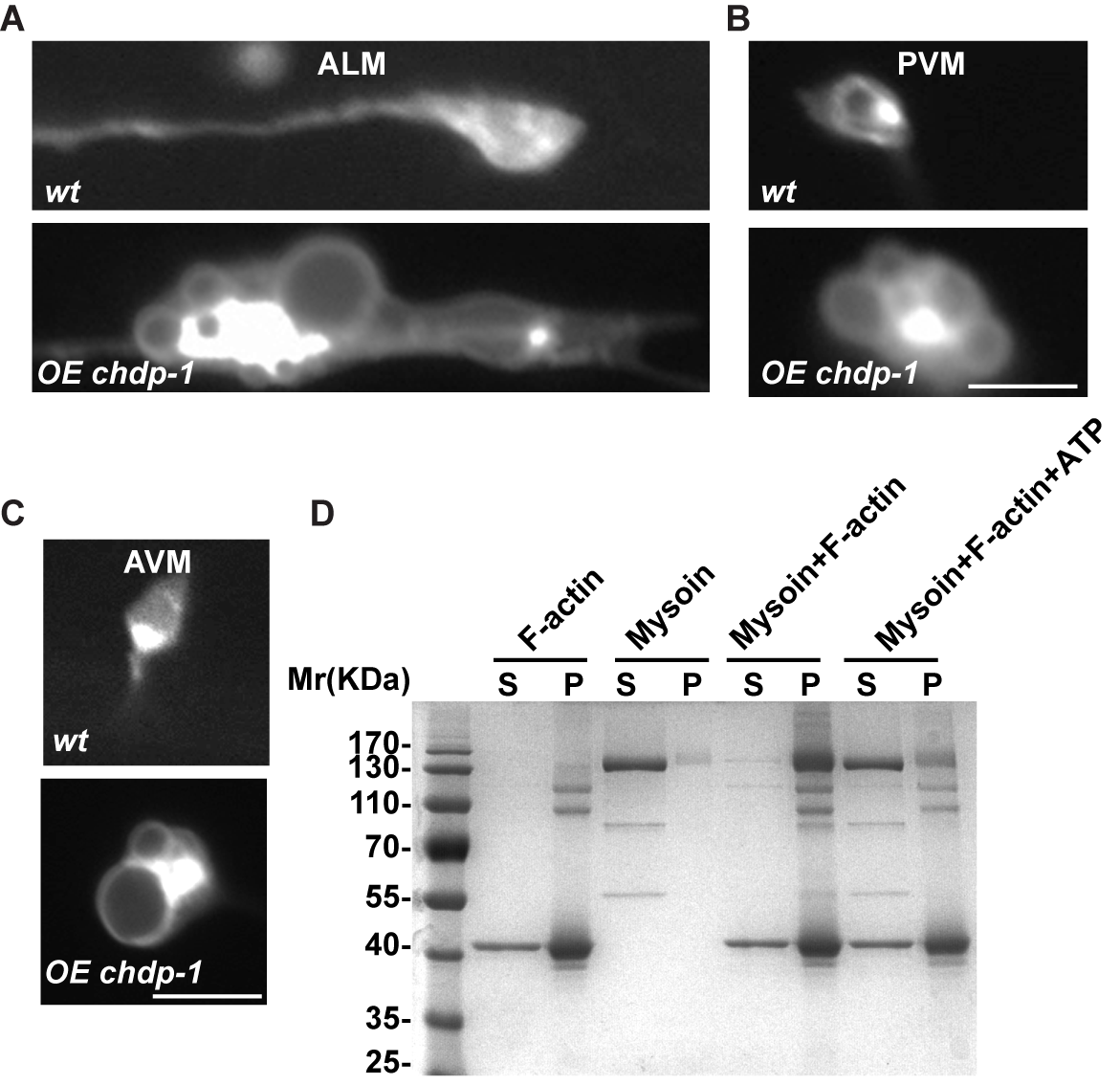

Supplement: S2 Fig — (A) Ectopic cell protrusions appear on ALM cells when chdp-1 is over-expressed (OE chdp-1). (B) Ectopic cell protrusions appear on PVM cells when chdp-1 is over-expressed (OE chdp-1). (C) Ectopic cell protrusions appear on AVM cells when chdp-1 is over-expressed (OE chdp-1). ALM, PVM and AVM are labeled by Punc-86::Myr::GFP. (D) The actin motor myosin co-sediments with F-actin and this myosin-actin association is disrupted by ATP. (TIF) [file pgen.1006163.s002.tif]

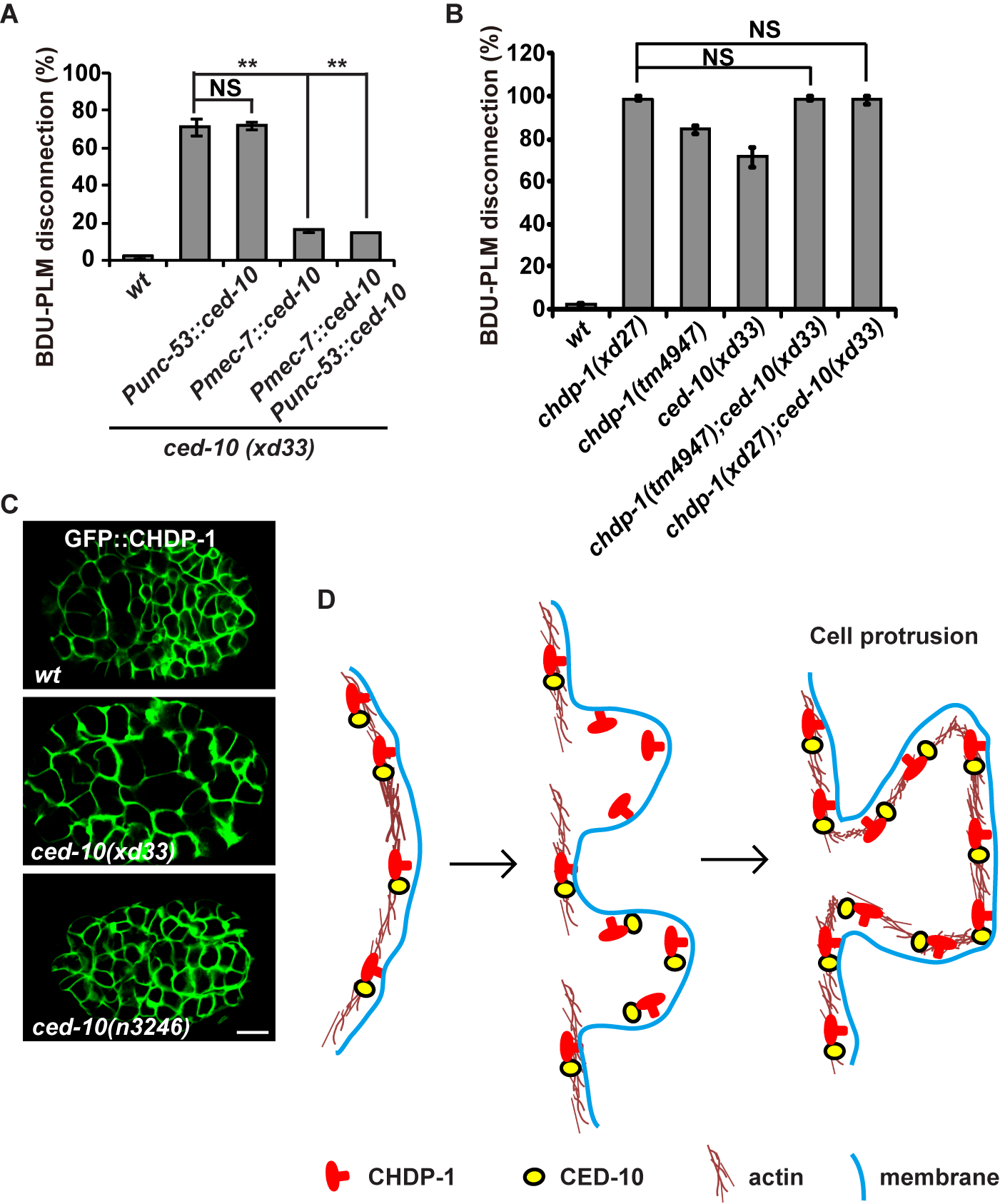

Supplement: S3 Fig — (A) Quantification of BDU-PLM connection defects in wild type and ced-10(xd33) mutants, and the corresponding rescuing strains. n ≥ 100. (B) Quantification of BDU-PLM connection defects in wild type, chdp-1(xd27), chdp-1(tm4947), ced-10(xd33) and the corresponding double mutant strains. n ≥ 100. (C) The membrane localization of GFP::CHDP-1 is not altered in ced-10(xd33) and ced-10(n3246) mutants. (D) Schematic drawing of the role of CHDP-1 and CED-10 in protrusion formation. (TIF) [file pgen.1006163.s003.tif]
