# Supplementary material for: The Calponin Family Member CHDP-1 Interacts with Rac/CED-10 to Promote Cell Protrusions
Source: PLoS Genet. 2016 Jul 14;12(7):e1006163. doi: 10.1371/journal.pgen.1006163 (PMC4944944; doi:10.1371/journal.pgen.1006163)
Supplement: S1 Table — (DOCX) [file pgen.1006163.s004.docx]

**S1 Table. Transgenes and strains**

| **Transgenes** | **DNA constructs** | **Host strains** |
| --- | --- | --- |
| *xdEx879*  *xdEx758* | *Pchdp-1:: GFP::CHDP-1*  *Pchdp-1::CHDP-1(C350T)::GFP* | *N2*  *N2* |
| *xdEx789* | *Pchdp-1::CHDP-1(C350T)* | *kyIs262* |
| *xdEx877* | *Pchdp-1:: CHDP-1Δhelix+ΔC::GFP* | *N2* |
| *xdEx1279* | *Pchdp-1:: CHDP-1Helix::GFP* | *N2* |
| *xdEx1281*  *xdEx1962*  *xdIs23*  *xdIs48* | *Pchdp-1:: CHDP-1ΔP1P2::GFP*  *Pchdp-1:: CHDP-1Δhelix::GFP*  *Pchdp-1::GFP*  *Pchdp-1::GFP::CHDP-1* | *N2*  *N2*  *N2*  *N2* |
| *xdEx910* | *Pchdp-1:: GFP::CHDP-1* | *chdp-1(xd27);kyIs262* |
| *xdEx1034* | *Pmec-7:: GFP::CHDP-1* | *N2* |
| *xdEx1337* | *Pmec-7::ACT-1::mCherry* | *N2* |
| *xdEx890*  *xdEx706* | *Pchdp-1:: CHDP-1*  *Pchdp-1::CHDP-1* | *chdp-1(tm4947);kyIs262*  *chdp-1(xd27);kyIs262* |
| *xdEx1297* | *Pmec-7:: CHDP-1* | *chdp-1(xd27);kyIs262* |
| *xdEx1301*  *xdEx929*  *xdEx931* | *Punc-53:: CHDP-1*  *Phyp-7::CHDP-1*  *Pmyo-3::CHDP-1* | *chdp-1(xd27);kyIs262*  *chdp-1(xd27);kyIs262*  *chdp-1(xd27);kyIs262* |
| *xdEx1474* | *Pmec-7::CED-10/Punc-53:: CED-10*  *Pmec-7::CED-10*  *Punc-53:: CED-10* | *ced-10(xd33);kyIs262* |
| *xdEx1475* |  | *ced-10(xd33);kyIs262* |
| *xdEx1477* |  | *ced-10(xd33);kyIs262* |
| *xdEx1619* | *Pmec-7:GFP:CED-10* | *N2* |
| *xdEx1635*  *xdEx1187* | *Pmec-7::GFP::CED-10/Pmec-7:RFP:CHDP-1*  *Pmec-7::GFP::chdp-1/Pmec-7::MYR::mcherry* | *N2*  *N2* |
| *xdEx1608* | *Pchdp-1:: CHDP-1* | *ced-10(xd33);kyIs262* |
| *xdEx1612* | *ced-10 fosmid* | *chdp-1(xd27);kyIs262* |
| *xdEx1568* | *Pchdp-1:: CHDP-1ΔP1* | *chdp-1(xd27);kyIs262* |
| *xdEx856* | *Pchdp-1:: CHDP-1ΔHelix+ΔC* | *chdp-1(xd27);kyIs262* |
| *xdEx893* | *Pchdp-1:: CHDP-1ΔCH* | *chdp-1(xd27);kyIs262* |
| *xdEx1616*  *xdEx1968*  *xdEx1964* | *Pchdp-1:: CHDP-1ΔC*  *Pchdp-1::chdp-1ΔHelix*  *Pmec-7::CHDP-1/Pmec-7::act::mCherry/Pmec-7::GFP::CED-10* | *chdp-1(xd27);kyIs262*  *chdp-1(xd27);kyIs262*  *N2* |
